# Maternal Choline Supplementation Alters Fetal Growth Patterns in a Mouse Model of Placental Insufficiency

**DOI:** 10.3390/nu9070765

**Published:** 2017-07-18

**Authors:** Julia H. King, Sze Ting (Cecilia) Kwan, Jian Yan, Kevin C. Klatt, Xinyin Jiang, Mark S. Roberson, Marie A. Caudill

**Affiliations:** 1 Division of Nutritional Sciences, Cornell University, Ithaca, NY 14853, USA; jhk288@cornell.edu (J.H.K.); sk2563@cornell.edu (S.T.K.); jy435@cornell.edu (J.Y.); kck68@cornell.edu (K.C.K.); xinyinjiang@brooklyn.cuny.edu (X.J.); 2 Department of Health and Nutrition Sciences, Brooklyn College, Brooklyn, NY 11210, USA; 3 Department of Biomedical Sciences, Cornell University, Ithaca, NY 14853, USA

**Keywords:** betaine, choline, fetal growth, placenta, pregnancy, IGF

## Abstract

Impairments in placental development can adversely affect pregnancy outcomes. The bioactive nutrient choline may mitigate some of these impairments, as suggested by data in humans, animals, and human trophoblasts. Herein, we investigated the effects of maternal choline supplementation (MCS) on parameters of fetal growth in a *Dlx3*+/− (distal-less homeobox 3) mouse model of placental insufficiency. *Dlx3*+/− female mice were assigned to 1X (control), 2X, or 4X choline intake levels during gestation. Dams were sacrificed at embryonic days E10.5, 12.5, 15.5, and 18.5. At E10.5, placental weight, embryo weight, and placental efficiency were higher in 4X versus 1X choline. Higher concentrations of hepatic and placental betaine were detected in 4X versus 1X choline, and placental betaine was positively associated with embryo weight. Placental mRNA expression of *Igf1* was downregulated by 4X (versus 1X) choline at E10.5. No differences in fetal growth parameters were detected at E12.5 and 15.5, whereas a small but significant reduction in fetal weight was detected at E18.5 in 4X versus 1X choline. MCS improved fetal growth during early pregnancy in the *Dlx3*+/− mice with the compensatory downregulation of *Igf1* to slow growth as gestation progressed. Placental betaine may be responsible for the growth-promoting effects of choline.

## 1. Introduction

The placenta mediates the delivery of nutrients to the fetus and is thus a major determinant of fetal growth [1]. Impairments in placental development can lead to placental insufficiency, an inability of the placenta to provide adequate nutrients for the fetus, and contribute to adverse pregnancy outcomes, including intrauterine growth restriction (IUGR), miscarriage, and preeclampsia [2,3]. At present, very few treatment options are available for placental-induced pregnancy complications that adversely affect the health of the mother and child. Nutritional therapies represent a promising area of research for improving maternal and child health [4], especially since some pharmacological treatments may be unsafe during pregnancy. Choline is a water-soluble essential nutrient that is necessary for the synthesis of acetylcholine (a neurotransmitter), phosphatidylcholine (a membrane phospholipid), and betaine (a methyl donor and osmolyte) [5]. During pregnancy, these molecules support rapid cell division, govern genomic methylation patterns, and modulate placental and fetal development [6].

Choline has previously been shown to reduce a risk factor of preeclampsia, sFLT1, in a randomized, controlled feeding trial of maternal choline supplementation (MCS) during the third trimester of pregnancy in healthy women [7]. In addition, choline has been shown to modulate inflammation, oxidative stress, and apoptosis of immortalized human placental trophoblast cells in culture [8] and in normal murine pregnancy [9]. Therefore, we hypothesized that choline supplementation would be beneficial for pregnancies complicated by placental insufficiency.

During pregnancy, the homeodomain-containing transcription factor distal-less homeobox 3 (*Dlx3*) plays a crucial role in the development of the fetal-maternal interface [10]. Mice heterozygous for this gene have been shown to display inadequate vascularization and development of the placental labyrinth, which is the location of nutrient exchange between mother and fetus [11]. Mice that are homozygous null for *Dlx3* die between embryonic days 9.5 and 12.5 due to placental failure and display extremely restricted fetal growth [10]. Heterozygous embryos are viable but have been reported to display placental abnormalities, including a reduced invasion of trophoblasts and impaired remodeling of maternal spiral arteries as well as increased placental oxidative stress and apoptosis within the labyrinth and maternal decidua [11]. 

A major determinant of early placental and fetal growth in both mice and humans is the insulin-like growth factor (IGF) axis, consisting of IGF1 and IGF2, their receptors IGF1R and IGF2R, and several binding proteins [12]. The placenta accumulates IGFs, IGF receptors, and IGF binding proteins, which help mediate the transfer of nutrients from placenta to fetus [13]. The IGF1 receptor transduces the growth-promoting function of IGFs, while the IGF2 receptor primarily functions to sequester and degrade IGF2 and prevent its binding to IGF1R. Accordingly, the genetic deletion of IGF1, IGF2, and IGF1R severely reduces fetal growth in mice, while the deletion of IGF2R leads to fetal overgrowth [14]. The expression of IGF genes as well as the epidermal growth factor receptor (EGFR), which interacts with IGF1R [15], has been shown to be modulated by choline supply in various tissues and gestational time points of pregnancy [16,17,18]. 

The present study was undertaken to investigate the effects of MCS on the determinants and parameters of fetal growth in the *Dlx3* murine model of placental insufficiency. We hypothesized that (a) maternal choline supplementation would support fetal growth in placental insufficiency and (b) this effect may occur through the IGF axis.

## 2. Materials and Methods

### 2.1. Mice and Diets

All animal protocols and procedures used in this study were approved by the Institutional Animal Care and Use Committees at Cornell University and conducted in accordance with the Guide for the Care and Use of Laboratory Animals (protocol number 2001-0034). The *Dlx3*+/− mice were a generous gift from Dr. Maria Morasso (NIH/NIAMS) and were genotyped using a three-primer duplex PCR (polymerase chain reaction) of tail DNA. The primers were designed to amplify wildtype or knockout allele amplicons or both (indicating a heterozygote) (Supplementary Table S1). The mice were housed in microisolator cages (Ancare) in an environmentally-controlled room (22–25 °C and 70% humidity) with a 12-h light-dark cycle. Breeding animals were given ad libitum access to commercial rodent chow (Teklad) and water. Female *Dlx3*+/− mice were mated with *Dlx3*+/− males. Female and male offspring were genotyped at time of weaning (three weeks of age), and *Dlx3*+/− animals were given ad libitum access to an AIN-93G purified rodent diet (Dyets #103345) containing 1.4 g choline chloride/kg diet (1X choline control diet). Five days before mating with *Dlx3*+/− males, *Dlx3*+/− females (*n* = 116) were randomized to receive either the 1X choline control diet (1.4 g/kg, the standard choline content of the AIN-93G diet providing adequate choline intake), a 2X choline diet containing 2.8 g choline chloride/kg (Dyets #103346), or a 4X choline diet containing 5.6 g choline chloride/kg (Dyets #103347). The 2X treatment was chosen to correspond with the previous human feeding study conducted by our research group [19]. A 4X treatment is widely used to investigate cognitive effects in rodent models. The presence of a vaginal plug was used to designate embryonic (E) day 0.5. Pregnant mice were euthanized at four different gestational time points (E10.5, E12.5, E15.5, and E18.5) in order to assess the effects of maternal choline supplementation across gestation.

### 2.2. Tissue Collection and Processing and Fetal Anthropometry

At the time of dissection, the maternal liver was removed, flash frozen in liquid nitrogen, and stored at −80 °C. The number of implantations and resorptions in the gravid uterus were recorded. Embryos and placentas were carefully dissected to minimize decidual contamination and weighed. At E12.5, E15.5, and E18.5, placentas were bisected across the chorionic plate; half was placed in RNA*later* for mRNA analysis, while the remaining half was immediately frozen in liquid nitrogen and stored at −80 °C for metabolite analysis. Due to the smaller tissue size, E10.5 placentas were alternately designated for mRNA analysis or metabolite analysis. Fetuses were imaged and subsequently frozen in liquid nitrogen and stored at −80 °C. The crown rump measurement of the fetuses was calculated from images using ImageJ software (NIH). Fetal DNA was extracted for *Dlx3* genotyping and sex determination using a commercial kit (Qiagen Inc., Germantown, MD, USA). Sex genotyping was performed by PCR for the *Sry* gene with a commercial kit (Qiagen Inc., Germantown, MD, USA). The primers are listed in Supplementary Table S1. 

Fetal body composition analyses were determined on fetuses acquired from E18.5 using the Leshner method [20]. Fetuses were decapitated at dissection to allow for analyses of the brain in a separate study; all body composition data is reported on the remaining tissue. Briefly, the fetuses were thawed and weighed, followed by desiccation to determine water weight. Dehydrated fetuses were weighed and pulverized before undergoing ethyl ether/methanol total lipid extraction. Total lipid extractions were successively performed until no change in weight occurred. The protein content was determined colorimetrically, following the digestion of pulverized fetal tissue in radioimmunoprecipitation assay (RIPA) buffer. 

### 2.3. Measurements of Hepatic and Placenta Metabolites

Concentrations of choline and the related metabolites betaine, methionine, phosphocholine, glycerophosphocholine, phosphatidylcholine, lysophosphatidylcholine, and sphingomyelin were measured in the maternal livers of dams by LC-MS/MS (liquid chromatography–tandem mass spectrometry) according to the method of Koc et al. [21], with modifications based on our equipment [19]. Choline, betaine, and methionine in the placenta were measured using the method of Holm et al. [22], with modifications based on our equipment [19].

### 2.4. Quantitative Real-Time RT-PCR

RNA was extracted from placentas fixed in RNA*later* using TRIzol reagent (Invitrogen, Waltham, MA, USA). Two or three placentas per dam were randomly selected for extraction. RNA concentration and quality were assessed with a NanoDrop ND-1000 instrument (Thermo Fisher Scientific, Waltham, MA, USA), and all samples had an A260/A280 ratio ≥1.8. Reverse transcription was performed using the ImProm-II Reverse Transcription System (Promega Corporation, Madison, WI, USA). Quantitative PCR was performed using SYBR^®^ Green in a Roche LightCycler480. All primers for the targeted genes (*Igf1*, *Igf2*, *Igf1r*, *Igf2r*, *Egfr*) were designed using NCBI Primer-BLAST (Supplementary Table S1). The reaction conditions were as follows: 95 °C for 5 min, followed by 40 cycles with 15 s at 95 °C, 30 s at 63 °C, and 30 s at 72 °C. A melting curve analysis was included at the end of the amplification cycles to ensure the specificity of the PCR product. Fold changes were calculated by the ΔC_t_ method [23] normalized to the expression level of housekeeping gene *Tbp* (TATA box binding protein). *Tbp* has been shown to be stable in response to various choline treatments [24]. 

### 2.5. Statistical Analysis

For maternal measurements, data were analyzed using general linear models with treatment and litter size as independent variables. Comparisons between embryonic genotype distributions were made using Fisher’s exact test with two-tailed P values. For placental and fetal measurements, data were analyzed using a linear mixed model. The model included choline treatment, fetal sex, and litter size as fixed effects and maternal identifier as a random effect. Data was either stratified by fetal *Dlx3* genotype or, when all genotypes were combined, was adjusted for fetal genotype as a fixed effect. Data were assessed for normality and log-transformed if the residuals were not normally distributed. Corrections for multiple analyses were not performed due to the hypothesis-driven nature of the study and the relatively limited sample size. Data are presented as mean ± SEM. *p* ≤ 0.05 was considered statistically significant, and 0.05 < *p* < 0.10 was considered to indicate trends. All analyses were performed using SPSS software, Version 23 (IBM).

## 3. Results

### 3.1. Effects of Maternal Choline Supplementation 

#### 3.1.1. Pregnancy Outcomes

To determine whether choline treatment during pregnancy could improve viability, we examined indicators of embryo survival. MCS did not significantly impact the number of implantations or the percentage of resorptions at any gestational time point in *Dlx3*+/− litters (Supplementary Table S2). We also assessed whether MCS could increase or prolong the survival of homozygous null fetuses at E10.5 and E12.5 (Supplementary Table S3). A small number of *Dlx3*−/− fetuses (*n* = 10) survived to E12.5, with the majority of these found in the 4X group (*n* = 7). Choline treatment at 2X or 4X did not significantly alter embryo genotype distributions at any gestational time point.

#### 3.1.2. Fetal and Placental Growth

**Figure 1:**
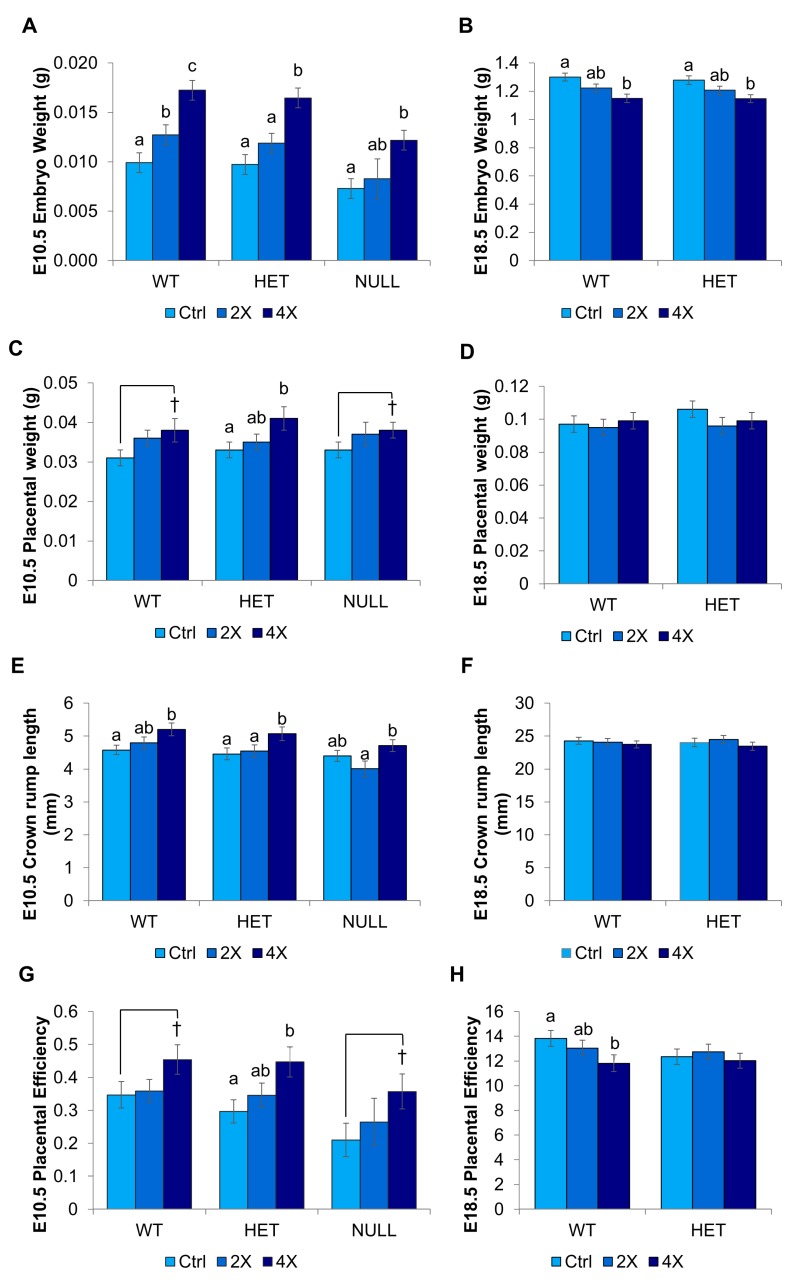
Fetal and placental growth characteristics by choline treatment (1X control, 2X, and 4X) and *Dlx3* genotype. Embryo weight at (**A**) E10.5, and (**B**) E18.5. Placental weight at (**C**) E10.5, and (**D**) E18.5. Crown-rump length at (**E**) E10.5 and (**F**) E18.5. Placental efficiency (defined as embryo weight/placental weight) at (**G**) E10.5 and (**H**) E18.5. Data were analyzed using mixed linear models controlling for maternal identifier, fetal sex, and litter size. Values are presented as mean ± SEM. Differing letters denote *p* ≤ 0.05. † denotes *p* < 0.10. *n* = seven to 10 dams per treatment per time point.

Since *Dlx3*+/− has been reported to be a model of placental insufficiency, we sought to determine whether MCS could improve placental efficiency as well as fetal growth. At E10.5, embryo weight was ~70% higher in 4X choline pups of all three genotypes compared to 1X controls (*p* < 0.001, 0.001, 0.033 for wildtypes, heterozygotes, and homozygous nulls, respectively). 2X (versus 1X) choline also yielded significantly higher embryo weights in wildtype pups (*p* = 0.038) (Figure 1A). At E18.5, the embryo weight of the 4X pups was slightly (but significantly) lower in both wildtype (~12%, *p* = 0.003) and heterozygote (~10%, *p* = 0.005) pups compared to 1X pups (Figure 1B).

Placental weight was significantly higher at E10.5 in 4X heterozygotes (~24%, *p* = 0.024 versus 1X) and tended to be higher in 4X wildtypes (~23%, *p* = 0.053 versus 1X) (Figure 1C). Placental weight was not influenced by choline treatment at E18.5 (Figure 1D). Fetal crown-rump length was ~14% higher at E10.5 in 4X choline wildtypes (*p* = 0.022) and heterozygotes (*p* = 0.033) compared to 1X controls but did not differ at E18.5 (Figure 1E,F). Placental efficiency, calculated as the ratio of embryo weight to placental weight, was ~51% higher at E10.5 in 4X choline heterozygotes (*p* = 0.016, vs. 1X) and tended to be higher in wildtypes (~31%, *p* = 0.088 vs. 1X) and homozygous nulls (~70%, *p* = 0.076 versus 1X) (Figure 1G). At E18.5, placental efficiency was ~15% lower in 4X wildtypes (*p* = 0.045 vs. 1X) but did not differ in heterozygotes (Figure 1H). 

At E12.5 and E15.5, the 2X and 4X choline groups of all genotypes did not significantly differ from 1X controls in embryo weight, placenta weight, crown-rump length, or placental efficiency (Supplementary Table S4). However, when comparing 2X to 4X, heterozygotes in the 4X group had slightly lower embryo weights at E12.5 (~19%, *p* = 0.011).

We also assessed the impact of altered growth patterns on fetal body composition at E18.5. Choline treatment at 2X or 4X did not alter the percentage of body water, fat, or protein compared to 1X choline (Supplementary Table S2). 

#### 3.1.3. Hepatic and Placental Choline Metabolites

Since the maternal liver is the source of choline metabolites, which are used by the placenta to support fetal growth and development, we measured the concentration of maternal hepatic choline metabolites (Table 1). At all four gestational time points, 4X (versus 1X) choline yielded significantly higher betaine concentrations by ~112%, ~114%, ~92%, and ~348% respectively (*p* < 0.001 for E10.5, 12.5 and 18.5; *p* = 0.001 for E15.5). 2X choline yielded higher betaine (~153% of 1X choline) at E18.5 (*p* = 0.022 vs. 1X). In the 1X control group, hepatic betaine concentration declined by ~35% from E10.5 to E18.5 (*p* = 0.042), whereas the 2X and 4X choline groups showed no significant change in hepatic betaine concentrations across gestation. 

At E12.5, glycerophosphocholine (GPC) was lower in both the 2X (~21%, *p* = 0.031) and 4X choline groups (~22%, *p* = 0.025) compared to the 1X choline group. No significant differences in GPC at E10.5, 15.5, or 18.5 were detected. At E15.5, phosphocholine was 133% higher in the 4X versus 1X choline group (*p* = 0.014). No significant differences in hepatic choline, methionine, phosphatidylcholine, lysophosphatidylcholine, and sphingomyelin were detected at any gestational time point (Table 1).

Placental concentrations of betaine were also higher in the 4X group at E10.5 (~39%, *p* = 0.007 vs. 1X) and tended to be 19% higher at E12.5 (*p* = 0.088 vs. 1X) (Table 2). However, betaine concentration did not differ by treatment in late pregnancy (E15.5 and E18.5). Placental concentrations of free choline and methionine were not significantly affected by choline treatment at any time point; however, methionine tended to be lower at E18.5 in the 4X group (~22%, *p* = 0.088, versus 1X).

#### 3.1.4. Placental Growth Factor Gene Expression

**Figure 2:**
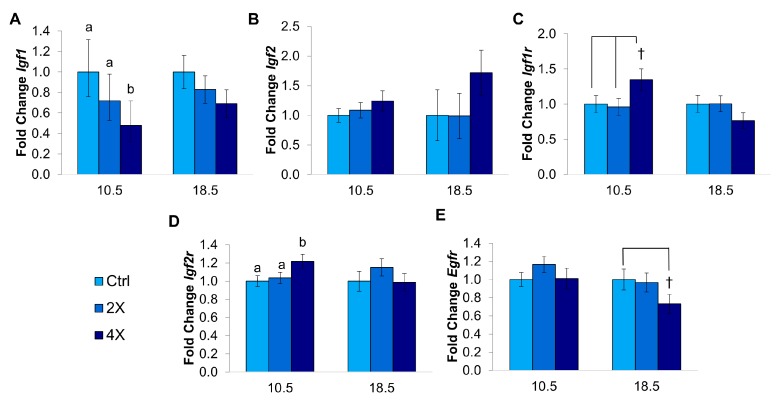
mRNA abundance of (**A**) *Igf1*, (**B**) *Igf2*, (**C**) *Igf1r*, (**D**) *Igf2r*, and (**E**) *Egfr* at E10.5 and E18.5 by maternal choline treatment (1X control, 2X, and 4X) in *Dlx3*+/− placentas. Fold changes are expressed relative to the housekeeping gene *Tbp*, with the 1X control group normalized to 1.0. Data were analyzed using mixed linear models controlling for maternal ID, fetal sex, and litter size. Log-transformed data (*Igf1* at E10.5) is represented by back-transformed means and 95% confidence intervals. All other values are presented as mean ± SEM. Differing letters denote *p* ≤ 0.05. † denotes *p* < 0.10. *n* = 20 placentas per treatment per time point (two to three per dam).

Since changes in fetal and placental growth were seen with choline treatment at E10.5 and E18.5, we measured the mRNA expression of several key growth-related genes in *Dlx3*+/− placentas at these time points. The placental expression of *Igf1* in the 4X (versus 1X) choline group was ~52% lower at E10.5 (*p* = 0.005) and, although not significant, was also lower (~34%) at E18.5 (Figure 2A). The placental expression of *Igf2* did not significantly differ at either time point (Figure 2B). *Igf1r* tended to have slightly higher expression (~35%) at E10.5 in the 4X choline group when compared to the 1X (*p* = 0.087) and 2X groups (*p* = 0.059) (Figure 2C). Similarly, the expression of *Igf2r* was higher (~22%) at E10.5 in the 4X groups compared to 1X (*p* = 0.035) (Figure 2D). Neither *Igf1r* nor *Igf2r* was significantly affected by choline treatment at E18.5. The expression of *Egfr* tended to be ~27% lower in the 4X choline treatment group at E18.5 versus the 1X controls (Figure 2E).

### 3.2. Effects of Dlx3 Genotype

#### 3.2.1. Fetal and Placental Growth

No significant differences in embryo weight, placenta weight, crown rump length, or placental efficiency were detected between wildtype and heterozygote pups at E10.5, 12.5, or 15.5. At E18.5, heterozygous pups weighed ~2% less (*p* = 0.044) and tended to have ~6% lower placental efficiency than wildtypes (*p* = 0.077) (Supplementary Figure S1A,B). Homozygous null pups weighed ~30% less than wildtypes and heterozygotes at E10.5 (*p* < 0.001) and ~59% less at E12.5 (*p* < 0.001) (Supplementary Figure S1C). They also had ~7% to 8% shorter crown rump lengths than wildtypes and heterozygotes at E10.5 (*p* < 0.001) and were ~23% to 25% shorter by E12.5 (*p* < 0.001) (Supplementary Figure S1D). Their placentas were ~31% to 32% less efficient than wildtypes and heterozygotes at E10.5 (*p* < 0.002) and ~52% to 53% less efficient at E12.5 (*p* < 0.001) (Supplementary Figure S1E). Homozygous null placentas did not differ in weight compared to wildtypes and heterozygotes at E10.5 (Supplementary Figure S1F). However, by E12.5, homozygous null placentas weighed ~17% less than those of wildtypes and heterozygotes (*p* = 0.027 and 0.019, respectively) (Supplementary Figure S1F).

#### 3.2.2. Growth Factor Gene Expression in the Placenta

Since we surprisingly observed similar fetal weights in *Dlx3* wildtype and heterozygous embryos, we also measured growth factor gene expression in E10.5 wildtype placentas to examine whether this could have resulted from compensatory changes in gene expression in *Dlx3*+/− placentas. Heterozygous pups overall had ~75% higher expression of *Igf1* compared to wildtypes (*p* = 0.002) (Supplementary Figure S2A). *Igf2*, *Igf1r*, and *Igf2r* expression did not differ between wildtype and heterozygous pups (not shown). Heterozygous placentas had ~43% higher expression of *Egfr* at E10.5 compared to wildtypes (*p* = 0.003) (Supplementary Figure S2B).

### 3.3. Determinants of Fetal Growth

#### 3.3.1. Choline Metabolites

**Figure 3:**
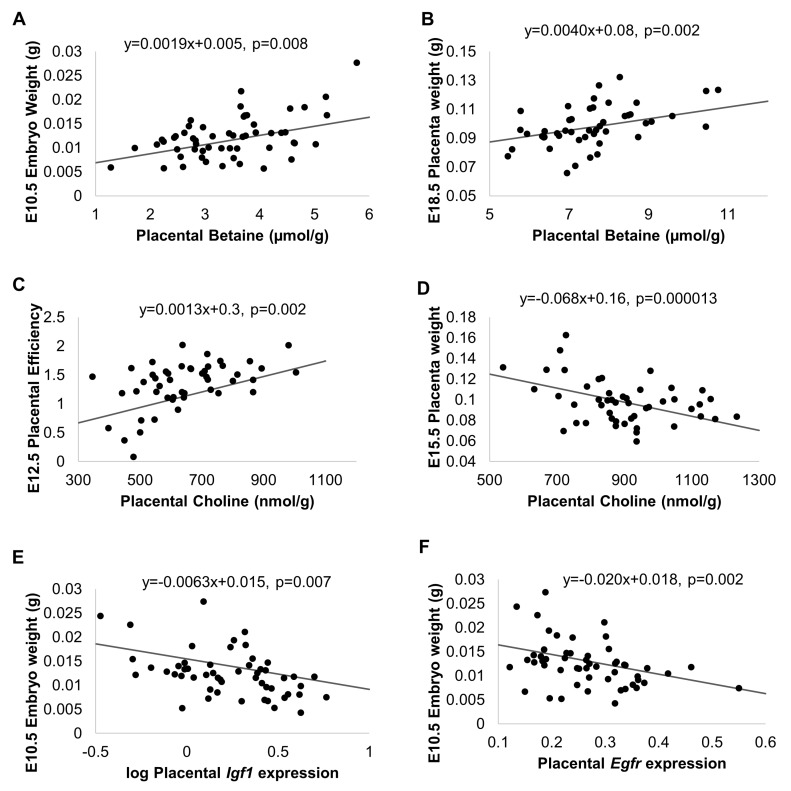
Relationships between placental choline metabolites, placental gene expression, and fetal and placental growth characteristics. Graphs present data points and regression lines obtained from linear mixed models adjusting for the mother’s ID, fetal genotype and sex, and litter size, with the intercept set at average litter size. (**A**) Placental betaine concentrations and embryo weight at E10.5; (**B**) Placental betaine and placental weight at E18.5; (**C**) Placental choline and placental efficiency at E12.5; (**D**) Placental choline and placental weight at E15.5; (**E**) Placental *Igf1* expression and embryo weight at E10.5; (**F**) Placental *Egfr* expression and embryo weight at E10.5.

To determine whether choline metabolites were associated with fetal growth, we performed regression analyses of placental metabolites and fetal and placental growth parameters, adjusting for choline treatment group, fetal genotype and sex, litter size, and maternal identifier (Table 3, highly significant relationships between metabolites and growth outcomes shown in Figure 3). Placental betaine at E10.5 was positively associated with embryo weight (*p* = 0.0082, Figure 3A) and crown rump length (*p* = 0.011) and tended to be positively associated with placental efficiency (*p* = 0.08). At E15.5, betaine was positively associated with embryo weight and placenta weight (*p* = 0.04, 0.0013, respectively). At E18.5, betaine was positively associated with placenta weight (*p* = 0.0015, Figure 3B) and negatively associated with placental efficiency (*p* = 0.01).

At E12.5, choline was positively associated with crown rump length (*p* = 0.041) and placental efficiency (*p* = 0.002, Figure 3C) and tended to be positively associated with embryo weight (*p* = 0.065) while being negatively associated with placenta weight (*p* = 0.0025). At E15.5, choline was negatively associated with embryo weight (*p* = 0.021) and placenta weight (*p* = 0.000013, Figure 3D). Choline tended to positively associate with crown rump length at E18.5 (*p* = 0.073). 

At E12.5, methionine was negatively associated with crown rump length (*p* = 0.01) and placental efficiency (*p* = 0.018) and tended to be negatively associated with embryo weight (*p* = 0.09). 

#### 3.3.2. Expression of Growth Factor Genes

We performed regression analyses to determine whether the expression of these growth factor genes was associated with our fetal and placental growth data, adjusting for choline treatment group, fetal genotype and sex, litter size, and maternal identifier (Table 4, highly significant relationships shown in Figure 3). At E10.5, placental *Igf1* expression was negatively associated with embryo weight and crown rump length (*p* = 0.007 and 0.043 respectively, Figure 3E). Similarly, *Egfr* expression was negatively associated with embryo weight, crown rump length, and placental efficiency (*p* ≤ 0.033 for all, Figure 3F). At E18.5, *Egfr* expression tended to be positively associated with embryo weight (*p* = 0.077), and *Igf2r* expression tended to be negatively associated with crown rump length (*p* = 0.099). Placental *Igf2* and *Igf1r* expression were not significantly associated with growth outcomes at either time point.

## 4. Discussion

In this study, we show for the first time that maternal choline supplementation modulates fetal growth in a model of placental insufficiency, the *Dlx3*+/− mouse. 

### 4.1. Supplementing the Maternal Diet with Extra Choline Accelerates Fetal Growth during the First Half of Pregnancy

Administering choline at 4X the recommended choline intake throughout pregnancy significantly increased embryo weight by mid-gestation (E10.5). This was accompanied by significant increases in placental weight, crown rump length, and placental efficiency in *Dlx3+/*− embryos and similar trends in *Dlx3*+/+ and −/− embryos. Notably, homozygous null fetuses at E10.5 in the 4X choline group achieved fetal weights and lengths similar to, or greater than, wildtype control diet embryos, suggesting a temporary rescue of the *Dlx3*−/− IUGR phenotype.

### 4.2. This Acceleration in Fetal Growth during Early Pregnancy Does Not Result in Overgrowth

By E12.5 and E15.5, no differences in placental or fetal size by choline treatment group were detected, suggesting that compensatory mechanisms had been engaged to attenuate the early acceleration in fetal growth. By late gestation (E18.5), a small (~12%) but significant decrease in embryo weight and placental efficiency was observed among the 4X (versus 1X) choline group. It is unlikely that the slightly smaller weight of 4X embryos compared to controls in the current study would have negative health consequences as this decrease did not coincide with any changes in body composition (fat or protein percentage). Additionally, the 4X embryo weights at E18.5 were similar to the control group embryo weights at E18.5 in a parallel study using a wildtype mouse with the same strain background under identical study conditions [9].

### 4.3. Supplementing the Maternal Diet with Extra Choline May Uniquely Benefit Pregnancies Characterized by Placental Insufficiency 

The acceleration in growth during early pregnancy in our *Dlx3* mice was not observed in a parallel study conducted by our research group in mice without placental insufficiencies. Maternal choline supplementation did not significantly affect embryo weight at any gestational time point in wildtype mice [9]. This suggests that providing extra choline may have a unique effect in mothers with compromised pregnancies who are at risk of IUGR. Since a growth-promoting effect was seen in both wildtype and *Dlx3+*/− fetuses, it appears that the mother’s genotype, rather than that of the fetus, is responsible. This may reflect the fact that circulating factors produced in dysfunctional placentas, such as sFlt-1, are capable of influencing their wildtype littermates. 

### 4.4. The Main Metabolic Fate of the Supplemental Choline in Our Study Was Betaine

Mothers in both choline groups had higher hepatic betaine levels at all four gestational time points. In control mothers, betaine was lower at E18.5 compared to all three earlier time points, suggesting a depletion of hepatic stores, which was prevented in the choline treatment groups. Placental betaine concentrations were also increased by 4X choline at E10.5 and E12.5. The positive association between placental betaine and embryo weight at E10.5, even controlling for choline treatment group, supports the role of betaine in promoting early fetal growth in the Dlx3+/− mouse. Notably, the placenta does not express the enzyme BHMT (betaine-homocysteine *S*-methyltransferase) and therefore cannot use betaine as a methyl donor. However, BHMT is expressed in the early embryo [25] and in fetal livers [26]. Thus, a possible explanation for improved fetal growth in early gestation may be the enhanced transfer of betaine from the placenta to the fetus, allowing its use of the methyl groups. Betaine may also reduce osmotic stress [27], which could have contributed to the observed improvements in placental efficiency. Nonetheless, inverse associations between circulating concentrations of betaine and birth weight have been reported in human pregnancy. Higher maternal circulating concentrations of betaine and higher umbilical cord betaine and choline have shown possible associations with lower birth weights [28,29]. However, one study also found a positive association between cord blood dimethylglycine, produced when betaine acts as a methyl donor, and birth weight [29]. This suggests that reduced flux through choline metabolic pathways in smaller babies may have resulted in higher circulating levels. 

### 4.5. Maternal Choline Supplementation also Altered Hepatic Phosphocholine and GPC Levels

Glycerophosphocholine (GPC) is an intermediate in the breakdown of phosphatidylcholine (PC); it has been shown to increase when demand for methyl groups is high, as in the case of folate deficiency [30]. At E12.5, mothers in both choline treatment groups had lower hepatic GPC levels, suggesting a reduced turnover of PC due to higher choline availability. GPC levels have been shown to be higher in preeclamptic placentas and to positively correlate with the preeclampsia risk factor sFLT1 [31], indicating that high PC turnover to regenerate methyl groups may be a characteristic of this disorder. Phosphocholine is an intermediate in the CDP-choline (cytidine diphosphate-choline) pathway; its conversion to CDP-choline is the rate-limiting step in PC synthesis [5]. At E15.5, 4X mothers had higher hepatic phosphocholine concentrations, potentially due to a larger supply of choline being directed into the CDP-choline pathway. 

### 4.6. Enhanced Early Growth Led to Placental down Regulation of Igf1

The growth promoting role of IGF1 has been demonstrated in knockout mouse models [32] and in mouse models of IUGR, whereby *Igf1* overexpression corrected the placental insufficiency and normalized fetal weight [33]. Human studies of IUGR pregnancies show a mixed relationship with IGF1, with some studies reporting higher maternal levels of IGF1 in IUGR pregnancies, suggesting compensatory upregulation in response to placental insufficiency [14]. Surprisingly, we found lower expression of *Igf1* in 4X choline placentas at E10.5. In addition, *Igf1* expression was inversely associated with embryo weight. The expression of *Egfr*, although not significantly altered by choline treatment, was also negatively associated with embryo weight, which was unexpected given that a lack of *Egfr* induces an IUGR phenotype [34]. These findings suggest that the observed downregulations of *Igf1* and *Egfr* are compensatory mechanisms that occurred in larger embryos to protect against excessive growth. A bidirectional relationship between placental *Igf1* expression and birth weight has been reported, whereby they are positively correlated in low and normal birth weights but inversely correlated in large babies [35]. In this way, *Igf1* may function to prevent extreme fetal weight in both directions. We also detected a slight but significant upregulation of *Igf2r* in 4X placentas at E10.5. As IGF2R regulates IGF2 availability by binding it and preventing signaling via IGF1R [36], this may represent an additional attempt by the placenta to restrain growth. 

With compensatory mechanisms already occurring at E10.5, it is likely that the mechanisms resulting in increased early growth occur before this time. IGF2 controls cell proliferation from E9 to E10, resulting in restricted growth by ~E11 if IGF2 is disrupted [37]. Therefore, it is possible that choline increased the expression of *Igf2* before E10.5. Although we did not detect significant upregulation of *Igf2* at E10.5, higher betaine availability combined with the activation of *Igf2* by methylation [38] supports this gene as a plausible candidate for early growth promotion. Alternatively, since the fetus, but not the placenta, is able to use betaine’s methyl groups, early modulation of the IGF axis may occur at the level of the fetus. Future studies examining gene expression in fetal tissues could assess this hypothesis. 

### 4.7. Dlx3+/− Placentas Had Higher Expression of Growth Factor Genes

Interestingly, *Dlx3*+/− placentas had higher expression of *Igf1* and *Egfr* at E10.5, a possible explanation for the similar weights of wildtype and *Dlx3*+/− embryos, in contrast to a previous study, which found reduced weights in heterozygotes compared to wildtypes [11]. Similar compensatory mechanisms via IGF1 have been reported in IUGR pregnancies [39,40]. Another possible explanation for the different study findings is the use of a purified AIN-93 diet in the current study (in contrast to a standard chow diet), which is specially formulated to support growth during pregnancy and may have allowed *Dlx3*+/− embryos to achieve normal weights. The survival of a subset of *Dlx3−*/*−* embryos at E12.5 in the 1X control group was unexpected and may be another reflection of the quality of the diet. Previously, *Dlx3−*/*−* embryos were detected at E12.5 only after treatment with TEMPOL, a strong antioxidant [11]. 

### 4.8. Strengths and Limitations

The strengths of our study include the use of four gestational time points, allowing a unique view of changes throughout pregnancy, and two supplemental doses, allowing the detection of dose-dependent effects. The use of an outbred mouse model displaying substantial variation between individuals limited our statistical ability to detect significant differences; however, it provided a wide range of responses to treatments, which is more similar to a human clinical trial and potentially leads to more generalizable findings. A disadvantage of using a mouse model to investigate pregnancy is that mouse fetuses are born at a comparatively underdeveloped stage compared to humans; thus, direct comparisons to pregnancy complications that occur late in gestation are difficult [41]. Our study was limited by the lack of a time point prior to E10.5, reducing our ability to determine mechanisms that increased early embryo growth. Additionally, our study was not powered to account for the effect of fetal sex; however, after determining that it was a source of variation in many outcomes, we included it in our statistical model. We were also limited by the high resorption rate and small litter sizes of *Dlx3*+/− mothers, which restricted our ability to perform further assays to assess protein expression and downstream signaling in the same cohort. 

## 5. Conclusions

In conclusion, maternal choline supplementation increased fetal and placental growth during early-mid gestation in a mouse model of placental insufficiency, resulting in compensatory changes to slow growth. The marked increase in embryo weight at mid gestation with choline treatment provides support for the potential use of choline as an intervention for IUGR, small for gestational age, and placental insufficiency. Since many pregnancy complications such as IUGR and preeclampsia increase the risk of early delivery [42], increasing fetal growth by mid gestation may avoid negative health outcomes for the fetus. In order to understand the future potential of maternal choline supplementation as a treatment in humans, further research is warranted to assess the effects in early gestation and determine the mechanisms by which choline and betaine impact fetal growth. Overall, these data show that, in this mouse model of placental insufficiency, supplementing the maternal diet with extra choline enhances fetal and placental growth in early-mid gestation without leading to major differences in later gestation.

## Figures and Tables

**Table 1:** Maternal liver concentrations of choline metabolites (per gram tissue) at E10.5, E12.5, E15.5, and E18.5 by choline treatment. Data were analyzed using general linear models with treatment and litter size as fixed factors. Values are presented as mean ± SEM. Log-transformed variables are represented by back-transformed means and 95% confidence intervals (in parentheses). * Significantly different versus control diet, *p* ≤ 0.05. ** Significantly different versus 1X and 2X diets, *p* ≤ 0.05. GPC, glycerophosphocholine; GPC, phosphatidylcholine; PCho, phosphocholine; LPC, lysophosphatidylcholine; SM, sphingomyelin. *n* = seven to 10 dams per treatment, per time point.

Time Point	Diet	Choline (nmol/g)	Methionine (nmol/g)	GPC (nmol/g)	LPC (nmol/g)	Betaine (µmol/g)	PCho (µmol/g)	PC (µmol/g)	SM (µmol/g)
10.5	1X	214 (173, 265)	124 (95, 161)	948 ± 105	332 (269, 411)	0.81 ± 0.1	1.03 ± 0.2	22.2 ± 0.9	3.94 ± 0.2
	2X	242 (186, 313)	92.7 (67, 127)	1021 ± 126	326 (252, 421)	1.49 ± 0.2 *	1.41 ± 0.2	21.6 ± 1.1	3.70 ± 0.2
	4X	247 (197, 310)	99.1 (75, 131)	895 ± 110	347 (277, 435)	1.71 ± 0.1 *	1.07 ± 0.2	22.2 ± 0.9	4.16 ± 0.2
12.5	1X	206 (173, 245)	85.5 (70, 104)	865 ± 54	363 (302, 437)	0.99 ± 0.1	1.08 (0.7, 1.6)	22.6 ± 0.7	3.97 ± 0.2
	2X	188 (156, 226)	108 (88, 133)	687 ± 57 *	353 (292, 429)	1.77 ± 0.2 *	1.20 (0.8, 1.8)	22.5 ± 0.7	4.03 ± 0.2
	4X	197 (166, 233)	97.5 (81, 118)	687 ± 52 *	371 (308, 446)	2.13 ± 0.1 *	1.38 (1.0, 2.0)	22.5 ± 0.7	3.75 ± 0.2
15.5	1X	138 (116, 163)	88.7 ± 5.9	294 (225, 382)	339 (259, 443)	1.21 ± 0.2	0.70 ± 0.2	22.4 ± 0.8	3.44 ± 0.2
	2X	133 (111, 160)	88.7 ± 6.4	337 (252, 449)	322 (245, 424)	1.60 ± 0.2 *	0.73 ± 0.3	21.6 ± 0.8	3.43 ± 0.2
	4X	144 (121, 171)	75.6 ± 6.0	262 (200, 344)	318 (243, 414)	2.32 ± 0.2 *	1.63 ± 0.3 *	22.3 ± 0.8	3.20 ± 0.2
18.5	1X	117 (97, 141)	104 ± 7.1	301 ± 34	286 (231, 355)	0.53 ± 0.2	0.58 ± 0.2	22.3 ± 1.0	3.10 ± 0.1
	2X	141 (116, 170)	83.3 ± 7.2	302 ± 34	262 (211, 325)	1.34 ± 0.2 *	1.04 ± 0.2	21.3 ± 1.0	3.23 ± 0.1
	4X	137 (113, 168)	97.4 ± 7.6	257 ± 36	252 (200, 316)	2.35 ± 0.3 **	0.91 ± 0.2	20.8 ±1.0	2.94 ± 0.1

**Table 2:** Placental concentrations of choline metabolites (µmol metabolite/g tissue) at E10.5, E12.5, E15.5, and E18.5 by choline treatment. Data were analyzed using mixed linear models with treatment, genotype, sex, and litter size as fixed effects and maternal ID as random effect. Values are presented as mean ± SEM. * Significantly different versus control diet, *p* ≤ 0.05. ** Significantly different versus 1X and 2X diets, *p* ≤ 0.05. # *p* < 0.1 vs. 1X diet. *n* = 20 placentas per treatment per time point (two to three per dam).

Time Point	Diet	Choline	Methionine	Betaine
E10.5	1X	0.50 ± 0.04	0.27 ± 0.02	3.09 ± 0.24
	2X	0.56 ± 0.04	0.30 ± 0.02	3.31 ± 0.27 *
	4X	0.52 ± 0.05	0.29 ± 0.03	4.28 ± 0.31 **
E12.5	1X	0.65 ± 0.06	0.25 ± 0.03	5.34 ± 0.50
	2X	0.62 ± 0.06	0.23 ± 0.03	5.59 ± 0.51
	4X	0.57 ± 0.05	0.25 ± 0.02	6.33 ± 0.42 #
E15.5	1X	0.90 ± 0.06	0.19 ± 0.02	7.99 ± 0.40
	2X	0.94 ± 0.06	0.21 ± 0.02	8.74 ± 0.43
	4X	0.87 ± 0.07	0.19 ± 0.02	8.27 ± 0.48
E18.5	1X	1.08 ± 0.08	0.20 ± 0.02	7.66 ± 0.46
	2X	1.14 ± 0.07	0.18 ± 0.02	7.42 ± 0.41
	4X	1.03 ± 0.08	0.16 ± 0.02 #	7.80 ± 0.42

**Table 3:** Placental concentrations of choline metabolites as determinants of placental and fetal growth characteristics at E10.5, E12.5, E15.5, and E18.5. Data were analyzed using individual mixed linear models with metabolite, treatment, genotype, and sex as fixed effects and maternal ID as a random effect. # *p* < 0.1, * *p* ≤ 0.05.

	E10.5		E12.5		E15.5		E18.5	
	β	*p* Value	β	*p* Value	β	*p* Value	β	*p* Value
**Embryo weight**								
Choline	0.0051	0.37	0.040	0.065 #	−0.171	0.021 *	−0.025	0.71
Betaine	0.0019	0.0082 *	−0.0011	0.61	0.018	0.040 *	0.0095	0.42
Methionine	−0.0032	0.75	−0.080	0.090 #	−0.45	0.11	0.018	0.95
**Placenta weight**								
Choline	−0.0055	0.64	−0.031	0.0025 *	−0.068	0.000013 *	−0.00058	0.94
Betaine	−0.00053	0.721	−0.00092	0.37	0.0059	0.0013 *	0.0040	0.0015 *
Methionine	−0.018	0.36	0.022	0.35	−0.072	0.25	−0.052	0.10
**Crown rump length**								
Choline	0.731	0.38	2.3	0.041 *	−0.65	0.53	2.1	0.073 #
Betaine	0.262	0.011 *	0.063	0.60	0.17	0.18	−0.064	0.76
Methionine	−0.012	0.99	−6.3	0.010 *	−3.1	0.43	2.7	0.60
**Placental efficiency**								
Choline	0.19	0.279	1.3	0.0020 *	0.33	0.63	−0.018	0.86
Betaine	0.042	0.080 #	−0.045	0.30	−0.017	0.84	−0.43	0.010 *
Methionine	−0.039	0.90	−0.23	0.018 *	−1.8	0.49	6.4	0.13

**Table 4:** Placental expression of growth-related genes as determinants of placental and fetal growth characteristics at E10.5 and E18.5. Data were analyzed using individual mixed linear models with gene fold change, treatment, genotype (for E10.5), and sex as fixed effects and maternal ID as a random effect. # *p* < 0.1, * *p* ≤ 0.05.

	E10.5		E18.5	
	β	*p* Value	β	*p* Value
Embryo weight				
*Igf1*	−0.0063	0.0072 *	−0.045	0.15
*Igf2*	0.00012	0.26	−0.00015	0.26
*Igf1r*	0.0021	0.55	0.072	0.12
*Igf2r*	−0.00025	0.84	−0.12	0.11
*Egfr*	−0.020	0.0019 *	0.14	0.077 #
Placenta weight				
*Igf1*	−0.0043	0.33	−0.00093	0.82
*Igf2*	0.070	0.71	0.000013	0.43
*Igf1r*	0.0084	0.20	−0.00033	0.59
*Igf2r*	−0.0014	0.51	−0.0014	0.14
*Egfr*	0.00065	0.096 #	−0.000080	0.99
Crown rump length				
*Igf1*	−0.64	0.043 *	−0.62	0.20
*Igf2*	−0.18	0.21	−0.0013	0.54
*Igf1r*	0.072	0.88	0.80	0.28
*Igf2r*	−0.17	0.33	−0.20	0.099 #
*Egfr*	−1.8	0.033 *	2.0	0.13
Placental efficiency				
*Igf1*	−0.88	0.24	−0.29	0.57
*Igf2*	0.0028	0.39	−0.00019	0.93
*Igf1r*	−0.10	0.36	1.1	0.17
*Igf2r*	−0.020	0.60	0.018	0.89
*Egfr*	−0.57	0.0041 *	1.3	0.36

## Supplementary Materials

The following are available online at www.mdpi.com/2072-6643/9/7/765/s1. Figure S1: Fetal and placental growth characteristics by *Dlx3* genotype, Figure S2: Growth factor gene expression by *Dlx3* genotype, Table S1: Primers for genotyping and RT-qPCR, Table S2: Litter size, resorptions, total implantations, and fetal body composition, Table S3: Genotype distributions, Table S4: Fetal and placental growth characteristics at E12.5 and E15.5.
