# Supplementary material for: Maternal Choline Supplementation Alters Fetal Growth Patterns in a Mouse Model of Placental Insufficiency
Source: Nutrients. 2017 Jul 18;9(7):765. doi: 10.3390/nu9070765 (PMC5537879; doi:10.3390/nu9070765)
Supplement: Supplementary file 1 [file nutrients-09-00765-s001.zip › nutrients-206532-Supplementary.pdf]

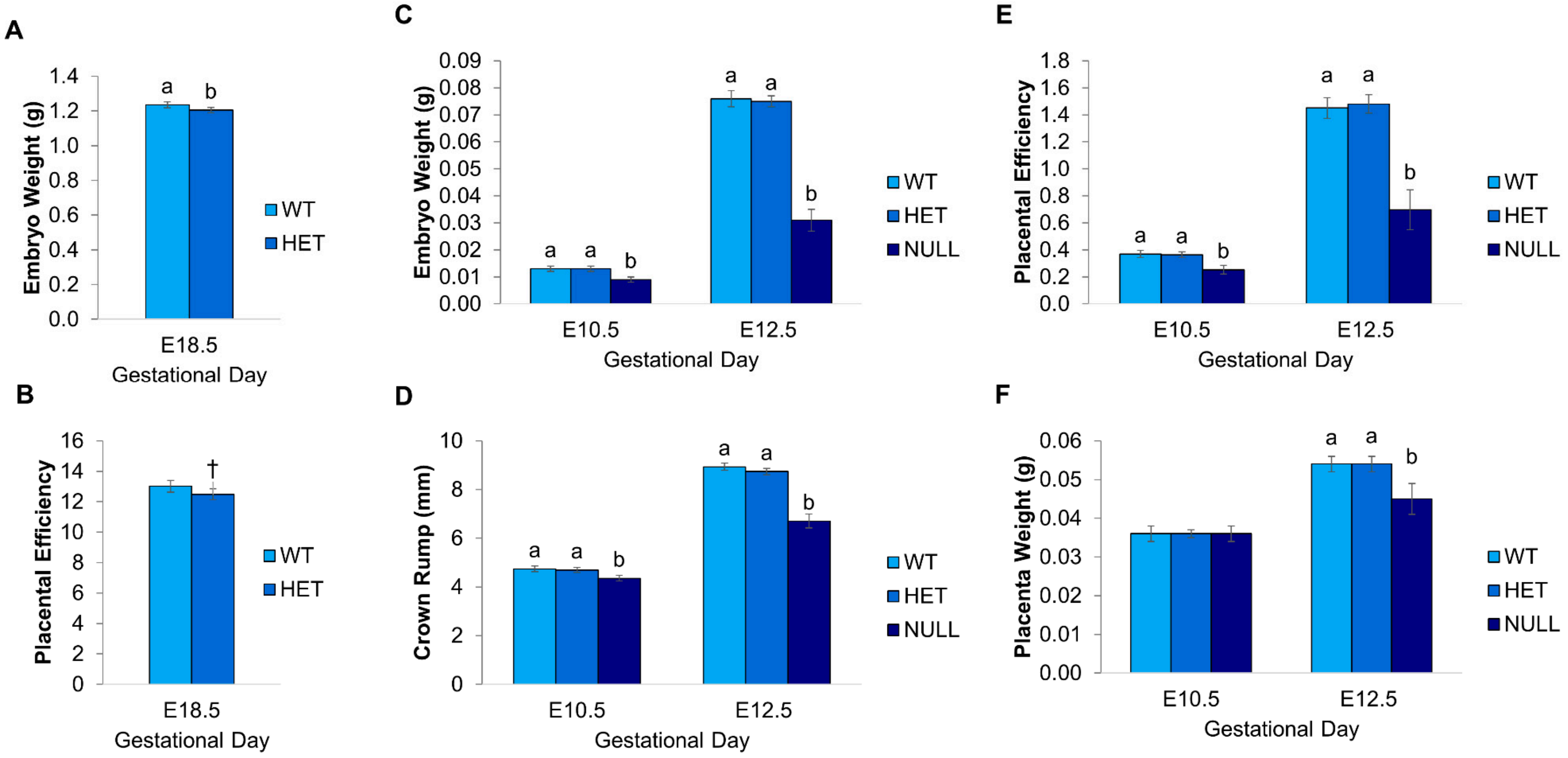

Supplementary Figure S1. Fetal and placental growth characteristics differ by *Dlx3* genotype. Embryo weight (A) and placental efficiency (B) at E18.5. Embryo weight (C), Crown rump length (D), placental efficiency (E), and placental weight (F) at E10.5 and E12.5. Placental efficiency defined as embryo weight/placental weight. Data were analyzed using mixed linear models controlling for choline treatment, maternal ID, fetal sex, and litter size. Values are presented as mean ± SEM. Differing letters denotes $P \leq 0.05$. † denotes $P<0.10$.

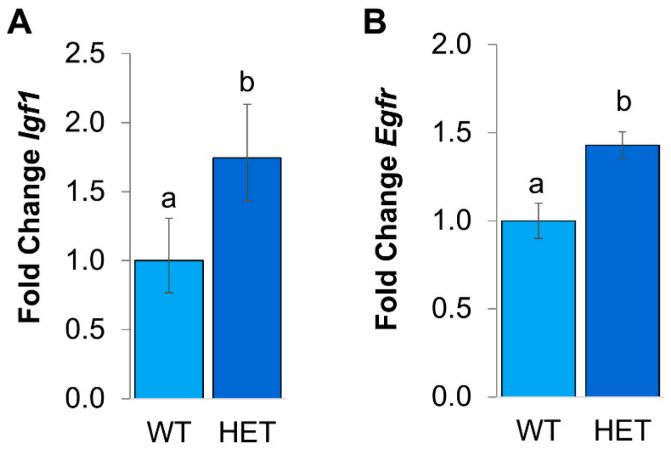

Supplementary Figure S2. Placental mRNA abundance of (A) *Igf1* and (B) *Egfr* at E10.5 by *Dlx3* genotype. Fold changes are expressed relative to the housekeeping gene *Tbp* with wildtypes normalized to 1. Data analyzed using mixed linear models controlling for choline treatment, maternal ID, fetal sex, and litter size. Log-transformed data (*Igf1*) is represented by back-transformed means and 95% confidence intervals. All other values are presented as mean ± SEM. Differing letters denotes P ≤ 0.05. † denotes P<0.10.

Table S1. Primers for genotyping and RT-qPCR.

Gene	*Name*	*Reference Sequence*	*Primer Sequences*	*Annealing Temperature*
Dlx3	Distal-less homeobox 3	NC_000077.6	F: 5′ GTGAACGGCAAGCCCAAA 3′<br>R: (Wild type allele)<br>5′ CTCTGTGACACGCCATACACAGTT 3′<br>R: (Knockout allele)<br>5′ AAAGGCCCGGAGATGAGGAAGAG 3′	Touchdown from 63° to 49°
Sry	Sex determining region Y	NC_000087.7	F: 5′ TGGGACTGGTGACAATTGTC 3′<br>R: 5′ GAGTACAGGTGTGCAGCTCT 3′	60°C
Igf1	Insulin-like growth factor 1	NM_010512.5	F: 5' GACCGAGGGGCTTTTACTTC 3'<br>R: 5' CATCCACAATGCCTGTCTGA 3'	63°C
Igf2	Insulin-like growth factor 2	NM_010514.3	F: 5' CGCTTCAGTTTGTCTGTTCG 3'<br>R: 5' GCAGCACTCTTCCACGATG 3'	63°C
Igf1r	Insulin-like growth factor receptor 1	NM_010513.2	F: 5' GCTTCGTTATCCACGACGATG 3'<br>R: 5' GAATGGCGGATCTTCACGTAG 3'	63°C
Igf2r	Insulin-like growth factor receptor 2	NM_010515.2	F: 5' TCTGTGTTGGCTCGTCACTC 3'<br>R: 5' CCGGTGACAGACGTTGATGA 3′	63°C
Egfr	Epidermal growth factor receptor	NM_207655.2	F: 5' GGACTGTGTCTCCTGCCAGAAT 3'<br>R: 5' GGCAGACATTCTGGATGGCACT 3′	63°C
Tbp	TATA-binding protein	NM_013684.3	F: 5′ AGGAGCCAAGAGTGAAGAACAA 3′<br>R: 5′ AACTTCACATCACAGCTCCCC 3′	60°C

Table S2. Litter size, resorptions, total implantations and fetal body composition in litters born to *Dlx3*+/- dams in response to three different maternal choline treatments (1X control, 2X and 4X) at E10.5, E12.5, E15.5 and E18.5. For litter size, implantations and % resorptions, data were analyzed using ANOVA. For body composition, data was analyzed using mixed linear models controlling for fetal genotype, maternal ID, fetal sex, and litter size and represented as % of total body weight. Values are presented as mean ± SEM.

Time point	Diet	Implantations	% Resorptions	Litter size	Fetal water content (%)	Fetal lipid content (%)	Fetal protein content (%)
**E10.5**	1X	12.3 ± 0.8	43.6 ± 5.7	7.2 ± 1.1			
	2X	12.4 ± 1.0	34.6 ± 6.9	8.3 ± 1.3	-	-	-
	4X	13.2 ± 0.9	47.6 ± 6.0	7.0 ± 1.1			
P (Treatment)		*0.722*	*0.731*	*0.371*			
**E12.5**	1X	10.6 ± 1.0	55.6 ± 6.3	5.1 ± 1.0			
	2X	10.2 ± 1.1	50.2 ± 6.9	5.3 ± 1.1	-	-	-
	4X	12.7 ± 1.0	50.6 ± 6.3	6.3 ± 1.0			
P (Treatment)		*0.631*	*0.198*	*0.804*			
**E15.5**	1X	11.9 ± 1.2	58.0 ± 5.1	4.9 ± 0.6			
	2X	10.6 ± 1.2	60.4 ± 5.1	3.8 ± 0.6	-	-	-
	4X	12.4 ± 1.2	51.9 ± 5.1	5.8 ± 0.6			
P (Treatment)		*0.100*	*0.549*	*0.491*			
**E18.5**	1X	12.8 ± 0.9	52.9 ± 7.8	5.9 ± 0.9	83.8 ± 0.35	0.91 ± 0.8	10.5 ± 1.2
	2X	11.0 ± 0.9	53.4 ± 7.4	4.8 ± 0.8	83.8 ± 0.33	1.87 ± 0.7	10.7 ± 1.1
	4X	12.1 ± 0.9	52.0 ± 7.8	5.6 ± 0.9	84.1 ± 0.34	1.27 ± 0.8	11.7 ± 1.2
P (Treatment)		*0.644*	*0.359*	*0.991*	*0.552*	*0.377*	*0.437*

Table S3. Genotype distributions in offspring born to *Dlx3*+/- dams in response to three different maternal choline treatments (control, 2X and 4X) at E10.5, E12.5, E15.5 and E18.5. 2-tailed P values; Fisher's exact test.

Time point	Diet		Embryo Genotype (%) WT	HET	NULL	P value (vs. Ctrl)
**E10.5**	1X		14 (21.5%)	35 (53.8%)	16 (24.6%)	
	2X		13 (25.0%)	33 (63.5%)	6 (11.5%)	0.20
	4X		10 (17.2%)	33 (56.9%)	15 (25.9%)	0.86
		Expected	*25%*	*50%*	*25%*	
**E12.5**	1X		23 (38.3%)	34 (56.7%)	3 (5.0%)	
	2X		21 (40.4%)	31 (59.5%)	0 (0.0%)	0.35
	4X		18 (24.3%)	49 (66.2%)	7 (9.5%)	0.17
		Expected	*33%*	*67%*	*0%*	
**E15.5**	1X		22 (40.0%)	33 (60.0%)	-	
	2X		15 (39.5%)	23 (60.5%)	-	1.0
	4X		24 (39.3%)	37 (60.7%)	-	1.0
		Expected	*33%*	*67%*	*0%*	
**E18.5**	1X		18 (34.0%)	35 (66.0%)	-	
	2X		22 (37.9%)	36 (62.1%)	-	0.70
	4X		15 (30.0%)	35 (70.0%)	-	0.68
		Expected	*33%*	*67%*	*0%*	

Table S4. Embryo weight, placental weight, crown rump length, and placental efficiency by *Dlx3* genotype at E12.5 and E15.5. Data were analyzed using mixed linear models controlling for maternal ID, fetal sex, and litter size. Values are presented as mean ± SEM. #*P* < 0.1 vs. 1X controls. **P* < 0.05 vs. 2X. n=7-10 dams per treatment, per time point. Values are presented as mean ± SEM.

Time point	Genotype	Diet	Embryo Weight	Placenta Weight	Crown Rump Length	Placental Efficiency
**E12.5**	**WT**	**1X**	0.080 ± 0.004	0.058 ± 0.003	9.00 ± 0.2	1.48 ± 0.1
		**2X**	0.076 ± 0.004	0.054 ± 0.003	8.69 ± 0.3	1.39 ± 0.1
		**4X**	0.071 ± 0.004	0.050 ± 0.003#	9.13 ± 0.2	1.49 ± 0.1
	**HET**	**1X**	0.076 ± 0.004	0.057 ± 0.003	8.89 ± 0.2	1.49 ± 0.1
		**2X**	0.083 ± 0.004	0.053 ± 0.003	8.76 ± 0.2	1.67 ± 0.2
		**4X**	0.068 ± 0.004*	0.052 ± 0.003	8.65 ± 0.2	1.35 ± 0.1
	**NULL**	**1X**	0.020 ± 0.007	0.037 ± 0.007	5.82 ± 1.0	0.68 ± 0.2
		**2X**	-	-	-	-
		**4X**	0.023 ± 0.004	0.047 ± 0.004	6.64 ± 0.4	0.50 ± 0.1
**E15.5**	**WT**	**1X**	*0.39* ± 0.02	0.098 ± 0.005	14.87 ± 0.4	4.14 ± 0.4
		**2X**	0.40 ± 0.03	0.084 ± 0.006	15.18 ± 0.3	4.84 ± 0.4
		**4X**	0.39 ± 0.03	0.095 ± 0.005	14.88 ± 0.3	4.23 ± 0.4
	**HET**	**1X**	*0.38* ± 0.02	0.094 ± 0.005	14.65 ± 0.2	4.11 ± 0.2
		**2X**	0.42 ± 0.03	0.087 ± 0.006	14.58 ± 0.3	4.80 ± 0.3#
		**4X**	0.39 ± 0.02	0.091 ± 0.005	14.91 ± 0.2	4.44 ± 0.2
